# Attentional bias in high math-anxious individuals: evidence from an emotional Stroop task

**DOI:** 10.3389/fpsyg.2015.01577

**Published:** 2015-10-19

**Authors:** Macarena Suárez-Pellicioni, Maria Isabel Núñez-Peña, Àngels Colomé

**Affiliations:** ^1^Department of Behavioural Sciences Methods, Faculty of Psychology, University of BarcelonaBarcelona, Spain; ^2^Institute for Brain, Cognition and Behaviour, University of BarcelonaBarcelona, Spain; ^3^Department of Basic Psychology, Faculty of Psychology, University of BarcelonaBarcelona, Spain

**Keywords:** attentional bias, emotional Stroop task, math anxiety

## Abstract

Attentional bias toward threatening or emotional information is considered a cognitive marker of anxiety, and it has been described in various clinical and subclinical populations. This study used an emotional Stroop task to investigate whether math anxiety is characterized by an attentional bias toward math-related words. Two previous studies failed to observe such an effect in math-anxious individuals, although the authors acknowledged certain methodological limitations that the present study seeks to avoid. Twenty high math-anxious (HMA) and 20 low math-anxious (LMA) individuals were presented with an emotional Stroop task including math-related and neutral words. Participants in the two groups did not differ in trait anxiety or depression. We found that the HMA group showed slower response times to math-related words than to neutral words, as well as a greater attentional bias (math-related – neutral difference score) than the LMA one, which constitutes the first demonstration of an attentional bias toward math-related words in HMA individuals.

## Introduction

Why do students with similar math ability choose alternative academic pathways at university? LeFevre et al. (1992) constructed a regression model to predict students’ choices of university majors varying in mathematical content and found that whereas age, fluency in math and experience with math contributed significantly to the choice, a “math affect” factor, comprising math anxiety and measures of avoidance toward math, more than doubled the variance accounted for by the model. Math anxiety has been defined as a feeling of tension, apprehension or even dread that interferes with the ordinary manipulation of numbers (Ashcraft and Faust, 1994). The negative effect of anxiety is reflected in poorer performance among high math-anxious (HMA) individuals (hereinafter, HMA), which, in turn, generates feelings of failure and, consequently, avoidance of this subject in the academic curriculum. As such, math anxiety leads people who are perfectly capable of doing math to distance themselves from mathematical contents and to feel afraid of the subject (for a recent review on the topic see Suárez-Pellicioni et al., 2015).

Although not recognized as a clinical condition, math anxiety is nonetheless a type of anxiety. Indeed, research has shown that findings related to other types of anxiety can be extended to the field of math anxiety. For example, as previously shown for generalized anxiety disorder or obsessive compulsive disorder (e.g., Gehring et al., 2000), a greater *error-related negativity* (i.e., an ERP component appearing approximately 150 ms after error commission) has been found in HMA individuals for errors committed in a numerical Stroop task but not in a control one (Suárez-Pellicioni et al., 2013b). Similarly, the reactive recruitment of attentional control observed for high trait anxious individuals (Osinsky et al., 2012) was also found for HMA ones, who exerted attentional control only after incongruent trials on a numerical Stroop task (Suárez-Pellicioni et al., 2014). Finally, several cognitive theories (Williams et al., 1988) have postulated that attentional bias toward threatening information can be considered a cognitive marker of numerous types of anxiety (Beck et al., 1985). In this respect, a wealth of research has confirmed that only anxious individuals display an attentional bias toward threatening information (Williams et al., 1996; Mogg and Bradley, 1998; Bar-Haim et al., 2007).

Similarly, the general theories trying to explain the negative effects of anxiety on performance have also been useful for explaining the negative effects of math anxiety on math performance. For example, the pioneering researchers on math anxiety (Ashcraft and Faust, 1994; Faust et al., 1996) interpreted their findings in the context of the *Processing efficiency theory* (PET; Eysenck and Calvo, 1992), one of the most important theories trying to explain the relationship between anxiety and performance in cognitive tasks. According to this theory, the anxiety reaction generates intrusive worrying thoughts that consume the limited attentional resources of the central executive of working memory (WM), which are then less available for task processing. Following this line, Ashcraft and Faust (1994) claimed that math anxiety affected performance only when complex –but not simple-arithmetic was involved and this effect would be due to HMA individuals devoting their WM resources to processing the worrying intrusive thoughts generated by the math anxiety reaction, instead of using them in solving the cognitive task.

In this line, this theory also claimed that anxiety affects *processing efficiency* (i.e., the relationship between the quality of performance and the amount of resources or effort needed to attain a given performance level) to a greater extent than *performance effectiveness* (i.e., quality of performance). In line with this theory, we found that although HMA and LMA participants did not differ in their level of performance in a simple addition verification task (i.e., no differences in performance effectiveness), the groups differed in processing efficiency, the HMA group investing more attentional resources (i.e., P600/P3b amplitude) than their LMA peers when a number far away from the correct solution (i.e., large-split) was presented as the proposed solution for the addition (Suárez-Pellicioni et al., 2013a).

However, the PET (Eysenck and Calvo, 1992) was questioned because of lacking precision and explanatory power, so a more recent theory, the *Attentional control theory* (ACT; Eysenck et al., 2007) emerged to improve those aspects. According to this theory, the specific function of WM affected by anxiety is attentional control, with anxiety causing an imbalance between the *stimulus-driven attentional system* (bottom-up) and the *goal-directed attentional system* (top-down). Given that HMA individuals would be more influenced by the former system, they would be more vulnerable to bottom-up attentional intrusions, that is, more vulnerable to distraction. In this respect, HMA individuals’ vulnerability to distraction was demonstrated by several studies, such as Suárez-Pellicioni et al. (2013a), who interpreted that this vulnerability would be at the base of HMA individuals’ difficulties in processing the above mentioned large-split solutions. More concretely, it has been considered that this imbalance between attentional systems would have its most detrimental effects on the inhibition function (Eysenck et al., 2007).

In this respect, several researchers have demonstrated that HMA individuals show greater difficulties to inhibit the influence of irrelevant information, such as reading non-italicized parts of a text (Hopko et al., 1998), or performing a numeric Stroop task in which participants have either to state the quantity of numbers while avoiding interference of numeric identity (i.e., 222222, correct answer, six; Hopko et al., 2002) or the number of greater numerical magnitude while avoiding interference of physical size (i.e., 2 8, correct answer, eight; Suárez-Pellicioni et al., 2014). Finally, the stronger influence of the stimulus-driven attentional system in high anxious individuals is also considered to be at the base of their tendency to preferentially allocate attentional resources to threat-related stimuli, as compared to neutral ones, generating an attentional bias toward this type of information (Eysenck and Byrne, 1992; Eysenck et al., 2007).

Attentional bias toward threat is considered to play an important role in the etiology and maintenance of anxiety disorders (e.g., Williams et al., 1996), by eliciting a “vicious cycle” where attention becomes hypervigilant to all the stimuli related to the person’s concerns or worries, which leads to a heightened emotional response (i.e., anxiety reaction). Thus, the greater sensitivity to these concerns would lead the individual to overestimate the level of danger in the environment or the degree of threat, aggravating their emotional disturbance. In this respect, MacLeod et al. (2002) administered medium-trait anxious individuals with a dot probe training procedure^1^ in order to establish a general disposition to attend selectively toward or away from emotionally negative information. They found that this attentional bias manipulation modified participants’ emotional responses to a stressful situation by influencing the degree to which they selectively processed different aspects of it (MacLeod et al., 2002), giving support to other studies proposing a causal role for attentional bias in anxiety conditions (see for instance Van den Hout et al., 1995).

Attentional bias has traditionally been measured with the emotional Stroop task, in which participants have to report the ink color of threatening (or emotionally charged) and neutral words presented in different ink colors (Williams et al., 1996). The emotional Stroop effect consists of a slower response time to threatening words than to neutral ones, which is considered to indicate the allocation of attention to emotional stimuli (processing word content instead of solving the main task of reporting ink color). The emotional Stroop task has been used successfully with patients with panic disorder (Dresler et al., 2012), specific phobia (Wikstrom et al., 2004), social phobia (Andersson et al., 2006), post-traumatic stress disorder (Ashley et al., 2013), generalized anxiety disorder (Mogg and Bradley, 2005), health anxiety (Karademas et al., 2008), etc. In non-clinical populations, the largest emotional Stroop effects are usually observed for those stimuli that relate to the participants’ current concerns, such as for dentist-related words for people showing anxiety toward dentist-related situations (Muris et al., 1995) or for cancer-related words in women with family histories of breast cancer (Erblich et al., 2003). Given the early mentioned parallelisms between math anxiety and other types of anxiety, would HMA individuals show an attentional bias as well? Would they be slower to report the ink color of math-related words as compared to neutral ones?

Two studies (Hopko et al., 2002; McLaughlin, unpublished thesis) have already tried to answer this question by means of the emotional Stroop task. First, in a study that used a paper version of the Stroop task including math-related and neutral words, McLaughlin (unpublished thesis) found no increase in response times to math-related words for HMA individuals. However, groups were formed using a split-half subject sample based on the mean math anxiety score, which means that the groups were not representative of extreme high and low math anxiety. Moreover, computer presentations of the task have been shown to be more powerful than the paper-and-pencil format for assessing Stroop-related effects (MacLeod, 1991). Given these methodological limitations, Hopko et al. (2002) decided to form the groups to be extreme on math anxiety scores (top and bottom 20% of their same-gender distribution). Furthermore, they used a computer-based version of the task in which each participant was presented with Stroop screens containing 100 words displayed in five different colors. Despite the authors’ efforts to overcome the methodological limitations of the study by McLaughlin (unpublished thesis), they still found no differences in response times, neither between groups nor between types of words. They acknowledged that this might have been due to the type of math-related words they used, which were probably too abstract (e.g., polynomial, theorem) and, therefore, less familiar to HMA individuals, who due to their math avoidance, tend not to enroll in advanced courses. Moreover, response times were calculated for each screen (i.e., 100 words), whereas calculating response times separately for each word would probably have been a more sensitive method.

Within this context, the objective of the present study was to demonstrate an attentional bias toward math-related words in HMA individuals, which would constitute the first step toward further investigation of this bias as a possible mechanism by which math anxiety may originate, be maintained and/or become aggravated. To achieve this objective we took steps to avoid the methodological limitations, which according to Hopko et al. (2002) might have prevented researchers from observing significant results in previous studies. Thus, we formed extreme groups and used a computer-based version of the task. In addition, we presented words individually in order to obtain a more accurate measure of response times, and we used more familiar math-related words. Moreover, we made sure that participants did not differ in trait anxiety, such that any differences between groups could not be explained by this variable. Finally, at the end of the experiment, participants were asked to provide a self-report measure of perceived anxiety to each stimulus.

## Materials and Methods

### Participants

Forty healthy volunteers were tested in this study, half of them with a high level of math anxiety (HMA) and the other half with a low level (LMA). They were selected from among a sample of 629 students from the University of Barcelona who were assessed for math anxiety and trait anxiety (see Materials and Methods) in the context of a larger project.

Participants were selected from the bottom quartile (LMA group) and from the top quartile (HMA group) of the Spanish version of the Abbreviated Mathematics Anxiety Rating Scale (sMARS; Alexander and Martray, 1989) scores. No participant was excluded from the study.

All participants had low scores on the Spanish version of the Zung Self-Rating Depression Scale (Conde et al., 1970; mean = 30.68, *SEM* = 1.03, range = 22–49), indicating that none of them should be classified as depressed.

Groups differed in math anxiety [*t*(38) = 19.90, *p* < 0.001] but not in trait anxiety [*t*(38) = 1.12, *p* = 0.26], depression [*t*(38) = 1.24, *p* = 0.22], age [*t*(38) = 0.25, *p* = 0.79], years of formal education [*t*(38) = 1.01, *p* = 0.31], handedness (χ^2^ = 0.36, *p* = 0.54), or ethnicity (χ^2^ = 1.02, *p* = 0.31). Groups also differed in gender distribution (χ^2^ = 7.03, *p* = 0.008), with more women in the HMA group. More detailed information about the two groups is shown in **Table 1**.

**Table 1:** Means and standard error of the mean (SEM; in brackets) for age, educational level, math anxiety, trait anxiety, and depression and frequencies for gender and manual dominance for the low math-anxious (LMA) and the high math-anxious (HMA) groups.

	Age	Gender	Dominance	Education	sMARS	STAI-T	Depression
LMA	21.95 (0.73)	9	19	9.40 (0.35)	44.95 (1.53)	16.95 (1.53)	29.40 (1.51)
HMA	21.70 (0.63)	17	18	9.90 (0.34)	86.40 (1.31)	20.15 (2.39)	31.95 (1.38)

*LMA, low math-anxious; HMA, high math-anxious; Gender, number of females. Dominance: number of right-handed; Education: number of years of formal education counting from 12 years-old forward. sMARS, Abbreviated Math Anxiety Rating Scale; STAI-T, Trait anxiety subscale from the STAI. Depression: Score at the Zung’s self-rating depression scale*.

Participants were paid for their participation, gave written informed consent before the experiment and were naïve as to the purposes of the study. All had normal or corrected-to-normal visual acuity and did not report any history of neurological or psychiatric disorders. The experimental protocol was approved by the Ethical Committee of the University of Barcelona.

### Materials

#### Screening Phase

Participants were administered the following instruments:

##### Shortened Mathematics Anxiety Rating Scale (Alexander and Martray, 1989)

The sMARS is a 25-item version of the Math Anxiety Rating Scale (MARS; Richardson and Suinn, 1972). This instrument measures math anxiety by presenting 25 situations which may cause math anxiety (e.g., *Being given homework assignments of many difficult problems that are due the next class meeting*). Items are answered on a five-point Likert scale, from 1 (no anxiety) to 5 (high anxiety). The possible total score therefore ranges from 25 to 125. The present study used the Spanish version of the sMARS (Núñez-Peña et al., 2013), which has shown strong internal consistency (Cronbach’s alpha = 0.94) and high 7-week test–retest reliability (intra-class correlation coefficient = 0.72).

##### State-Trait Anxiety Inventory (STAI)

Only the trait anxiety subtest was used. This includes 20 statements describing different emotions. Respondents have to answer by considering how they feel ‘in general’. Items are answered on a four-point Likert scale, with options ranging from 0 (almost never) to 3 (almost always). Good to excellent internal consistency (Cronbach’s alpha = 0.89–0.96) and adequate 30-day test–retest reliability (*r* = 0.75) have been reported with high-school students (Spielberger et al., 1983). The Spanish version of this test, which has also shown good psychometric properties (Spielberger et al., 2008), was used in this study.

### Experimental Session: Pretest

Participants were administered the following scale:

#### Zung Self-Rating Depression Scale

This scale contains 20 statements. Respondents have to rate the items according to how they apply to him/her over the last few days, using four response options reflecting the frequency of occurrence. Total scores range from 20 to 80, and a score below 49 is considered to indicate no depression. The present study used the Spanish version of this test (Conde et al., 1970), which shows good internal consistency (Cronbach’s alpha = 0.79–0.92) and good validity evidence (correlation with the Hamilton and Beck depression scales ranging from 0.50 to 0.80).

### Experimental Session: The Emotional Stroop Task

Fourteen neutral words and 14 math-related words were used in the experiment (stimuli are listed in the Appendix). The words were obtained through a questionnaire administered to 117 year-two students from the Faculty of Psychology of the University of Barcelona. This questionnaire asked participants to write down the first 15 words that came to mind when thinking about mathematics. From this information we selected the 14 words that were most reported by students as being math-related. We then selected 14 neutral words from the Spanish lexical database of NIM (Guasch et al., 2013; http://www.bcbl.eu/databases/espal/) that matched the math-related words on several characteristics. Consequently, words in the two categories did not differ in frequency [*t*(26) = 0.02, *p* = 0.97], number of phonemes [*t*(26) = 0.08, *p* = 0.93], familiarity [*t*(22) = 0.38, *p* = 0.70], imageability [*t*(22) = 1.04, *p* = 0.30], or concreteness [*t*(22) = 0.71, *p* = 0.48]^2^. **Table 2** shows more detailed information about words characteristics.

**Table 2:** Mean and standard error of the mean (in brackets) for neutral and math-related words’ characteristics.

	Neutral words	Math-related words
Frequency	46.58 (23.05)	47.55 (24.19)
Number of phonemes	8.28 (0.52)	8.35 (0.72)
Familiarity	5.30 (0.16)	5.41 (0.22)
Imageability	4.90 (0.16)	4.54 (0.34)
Concreteness	4.73 (0.09)	4.62 (0.12)

The two types of words were presented in separate blocks, that is, a set of math-related words and another set of neutral words. According to Bar-Haim et al.’ (2007) meta-analysis, blocked presentation of stimuli produced a significantly larger combined effect size as compared to randomized presentations (see also Holle et al., 1997). Indeed, the emotional Stroop effect in healthy participants is considered to be a rather slow effect that builds up over subsequent trials (i.e., a carryover effect; McKenna and Sherma, 2004; Phaf and Kan, 2007), the cumulative exposure to threat-related stimuli probably being at the base of stronger perceived threat as compared to randomized presentations. Each block included 58 stimuli: 2 fillers (excluded from the analysis) followed by 56 stimuli corresponding to the 14 words presented in the four ink colors. Stimuli in each block were presented pseudo-randomly, with the only restriction being that the same ink color was never presented in two consecutive trials. Blocks were presented in counterbalanced order and were separated by one minute rest.

The E-prime 2.0 program (Psychology Software Tools Inc., Sharpsburg, PA, USA) was used to control the presentation and timing of the stimuli and the measurement of response accuracy and response times.

### Experimental Session: Post-test

At this point, participants were administered the self-report questionnaire, which asked them to rate the level of anxiety generated by each word. There were five response options, ranging from 1 (*Nothing*) to 5 (*A lot*). Participants were told to respond by taking into account their thoughts and feelings while performing the emotional Stroop task.

### Procedure

Participants were tested individually. Upon entering the experimental room, they completed standard procedures concerning informed consent along with a demographics questionnaire asking their age, manual dominance, gender, and number of years of formal education. Participants were tested individually. After that, they were administered with the Zung’s self-rating depression scale (Zung, 1965). Then, participants were given detailed task instructions.

The session began with a training block of 20 words, all of them neutral and different from the ones presented in the experimental session (e.g., Franken et al., 2009). When participants achieved 65% of hits in the training period, the experimental session started. The training trials were only used to familiarize the participants with the task, so they were excluded from the statistical analysis.

Stimuli were presented at the center of a black screen in font type Tahoma (size 35; lowercase) and in four different ink colors (red, blue, green, and yellow). The task for participants consisted in responding to the ink color of the stimuli by means of a button press, as fast and as accurately as possible. Participants responded with the index and middle finger of each hand, using a keyboard and setting their fingers on the response buttons. Response buttons were color-coded with a sticker so that “red”, “blue”, “green”, and “yellow” responses corresponded, respectively, to the letters “d”, “f”, “j”, and “k” on the keyboard. Each trial began with a fixation sign (an asterisk) shown for 500 ms. After that, a word was presented on the screen and remained there until a response was given (maximum of 1500 ms). Each trial was followed by a variable inter-trial interval ranging from 1000 to 1600 ms (a black screen).

## Data Analysis and Results

### Behavioral Measures

Means of response times were calculated for correctly solved trials for each condition and for each participant. Means were calculated after eliminating outliers according to Tukey’s method (Tukey, 1977). In this method, extreme outliers are defined as greater or equal to 3 interquartile ranges above the upper quartile (Q3) (i.e., extremely high values) and slower or equal to 3 interquartile ranges below the lower quartile (Q1) (i.e., extremely low values). More concretely, we started by performing boxplots for the response time scores for each participant. Then, we eliminated those values identified as outliers, that is, those that were shown as dots outside the range of the whiskers. Finally, we calculated means of response times for each participant in each condition without the influence of those extreme values. Thus 2.92% of all trials were discarded (2.99% for the LMA group and 2.85% for the HMA one). Percentages of hits were also calculated for each participant in each condition. Response times and percentage of hits were analyzed through analyses of variance (ANOVAs), taking *Stimuli* (math-related word and neutral word) as the within-subject factor and *Group* (LMA and HMA) as the between-subjects factor. The *F* value, the uncorrected degrees of freedom, the probability level following correction, the ε value (when appropriate), and the partial eta square index ($\eta_{p}^{2}$) are given. We performed tests of simple effects when an interaction was significant, and used the Bonferroni correction to control for the increase in Type I error.

Moreover, a single score of attentional bias was calculated by subtracting the neutral condition from the math-related one, both for response times and for hit rates. For response times, the greater the index, the greater the attentional bias (i.e., more time needed to respond to math-related words than to neutral ones). As for percentage of hits, the slower the index, the greater the attentional bias (i.e., more errors are committed when responding to math-related words than for neutral ones). Student *t*-tests were carried out to compare this index between groups.

**FIGURE 1:**
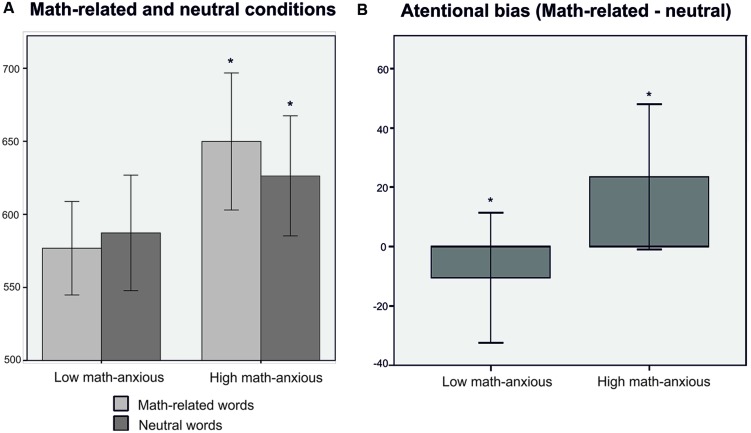
**Means and standard errors (in bars) for response times (in ms) for the low math-anxious (LMA) and high math-anxious (HMA) groups when responding to neutral and math-related words **(A)** and for the attentional bias **(B)**.** Significant differences at ^∗^*p* < 0.05.

Regarding response times, we found a significant main effect of *Group* [*F*(1,38) = 4.67, *p* = 0.03, $\eta_{p}^{2}$ = 0.11], with the HMA group being slower than the LMA one. More interestingly, we found a significant *Stimuli* × *Group* interaction [*F*(1,38) = 4.28, *p* = 0.04, $\eta_{p}^{2}$ = 0.10]. Simple effects analyses showed that the HMA group took longer to respond to math-related words than to neutral ones [*t*(19) = 1.92, *p* = 0.050, effect size *r* = 0.40], whereas no difference emerged for the LMA group [*t*(19) = 0.95, *p* = 0.37, effect size *r* = 0.21]. On the other hand, when comparing groups for each condition we found that groups differed when responding to math-related words [*t*(38) = 2.69, *p* = 0.01, effect size *r* = 0.39], with the HMA group being slower than the LMA one; however, this group difference was not observed when responding to neutral words [*t*(38) = 1.43, *p* = 0.16, effect size *r* = 0.22]. Moreover, groups differed on the attentional bias index (math-related – neutral) [*t*(38) = 2.07, *p* = 0.04], the HMA group showing greater attentional bias than their LMA peers. Response times for math-related and neutral words (A) and for the attentional bias index (B) for each group are shown in **Figure 1**.

Regarding the percentage of hits, no main effects or interaction reached significance (all *p*-values above 0.25). Similarly, groups did not differ in the attentional bias index [*t*(38) = 1.21, *p* = 0.23]. Means and SEM for response times and percentage of hits for each group and for each stimulus are shown in **Table 3**.

**Table 3:** Means of RT (SEM in brackets), percentage of hits and self-reported measures of anxiety for math-related words, neutral words and for their attentional bias index (math-related – neutral) for the LMA and HMA groups.

	LMA			HMA		
	Math-related	Neutral	Attentional bias	Math-related	Neutral	Attentional bias
RT	576.83 (15.28) •	587.32 (18.89)	-10.48 (10.95) •	649.89 (22.41) ∘•	626.36 (19.63) ∘	23.52 (12.23) •
Accuracy	94.50 (0.82)	93.14 (0.89)	1.35 (0.90)	93.90 (0.82)	94.00 (0.89)	0.10 (0.89)
Self-reported	15.85 (0.87) •	15.65 (1.00)	0.20 (0.43) •	31.05 (2.44) ∘•	16.65 (0.92) ∘	14.40 (2.28) •

*∘, Significant differences between conditions; •, Significant differences between groups*.

### Words’ Anxiety Ratings

An ANOVA was performed taking *Stimuli* as the within-subject factor and *Group* as the between-subjects factor. The ANOVA showed a significant *Stimuli* × *Group* interaction [*F*(1,38) = 37.23, *p* < 0.001, $\eta_{p}^{2}$ = 0.49]: specifically, the HMA group reported a higher level of anxiety for math-related words as compared with neutral words [*t*(19) = 6.28, *p* < 0.001], whereas no such difference was observed for the LMA group [*t*(19) = 0.46, *p* = 0.90]. When stimuli assessment was compared across groups, they were found to differ for math-related words [*t*(38) = 5.86, *p* < 0.001], but not for neutral words [*t*(38) = 0.73, *p* = 0.47], with the HMA group reporting higher levels of anxiety than the LMA group. In order to be consistent with the analysis of response times and hit rates, a difference score was calculated by subtracting the anxiety reported toward neutral words from the one reported toward math-related ones. This analysis showed that groups differed in this index [*t*(38) = 6.10, *p* < 0.001], showing greater difference for the HMA group than for the LMA one. Means and SEM for these self-reported measures are shown in **Table 3**.

### Relationship among Response Times, Words’ Anxiety Ratings and Level of Math Anxiety

Participants’ levels of math anxiety, trait anxiety, depression and years of formal education were correlated with behavioral (response times and percentage of hits) and self-reported measures to math-related and neutral words, as well as for the attentional bias score (math-related – neutral) in order to further our understanding of the relationship among these variables.

As shown in **Table 4**, results showed significant positive correlations between the sMARS and the time needed to respond to math-related words (*r* = 0.38, *p* = 0.01) and with the math-related words ratings (*r* = 0.70, *p* < 0.001) and for the self-reported difference score (*r* = 0.69, *p* < 0.001). On the contrary, no significant correlations emerged between the sMARS scores and the time needed to respond to neutral words (*r* = 0.23, *p* = 0.13) or with the neutral words ratings (*r* = 0.16, *p* = 0.30).

**Table 4:** Pearson correlation coefficients between subject variables, behavioral (response times and accuracy) and self-reported measures for math-related words, neutral words and their difference (attentional bias) for the whole sample (*n* = 40).

		Subject variables				Response times			Accuracy			Self-reported measures		
		sMARS	STAI-R	Depr	Educ level	Math	Neutral	AttBias	Math	Neutral	AttBias	Math	Neutral	AttBias
**Subject** **variables**	sMARS		0.19	0.12	0.10	0.38^∗^	0.23	0.27	-0.03	0.10	-0.14	0.70^∗∗^	0.16	0.69^∗∗^


	STAI-R			0.59^∗∗^	-0.03	0.42^∗∗^	0.39^∗^	0.08	-0.09	0.04	-0.12	0.39^∗∗^	0.40^∗∗^	0.26
	Depr				0.23	0.27	0.12	0.25	-0.08	0.16	-0.24	0.18	0.24	0.09
	Educ level					0.09	0.23	-0.19	-0.06	0.30	-0.36^∗^	0.01	-0.07	0.04
**Response times**	Math						0.81^∗∗^	0.40^∗∗^	-0.50	0.31^∗^	-0.35^∗^	0.47^∗∗^	0.07	0.48^∗∗^


	Neutral							-0.20	0.03	0.30	-0.27	0.31^∗^	0.05	0.32^∗^
	AttBias								-0.13	0.04	-0.16	0.30	0.04	0.30^∗^
**Accuracy**	Math									0.44^∗∗^	0.46^∗∗^	-0.15	0.08	-0.20
	Neutral										-0.58^∗∗^	-0.05	-0.20	0.02
	AttBias											-0.08	0.28	-0.21
**Self-reported measures**	Math												0.40^∗∗^	0.92^∗∗^


	Neutral													0.02
	AttBias													

*^∗^*p* ≤ 0.05; ^∗∗^*p* ≤ 0.01; Depr, Depression; AttBias, math-related – neutral; Accuracy, Percentage of hits*.

Interestingly, a positive significant correlation emerged between trait anxiety and behavioral measures, so the higher the level of trait anxiety the slower the response times for both math-related (*r* = 0.42, *p* = 0.006) and neutral (*r* = 0.39, *p* = 0.01) words, and the higher the self-reported measures of anxiety for both math-related (*r* = 0.39, *p* = 0.01) and neutral (*r* = 0.40, *p* = 0.008) words.

As for the relationship between response times and the self-reported level of anxiety generated by words, first, a significant positive correlation emerged between the response times for math-related words and the anxiety ratings for them (*r* = 0.47, *p* = 0.002), so the greater the anxiety reported, the slower the response to them. On the contrary, the time needed to respond to neutral words showed a non-significant correlation with the anxiety ratings for these words (*r* = 0.05, *p* = 0.73). The same positive correlation emerged for the response times and self-reported difference scores (*r* = 0.30, *p* = 0.04), so the higher the difference in response times (i.e., more time needed to math-related words as compared to neutral ones), the higher the self-reported levels of anxiety generated by math-related words as compared to neutral ones.

## Discussion

This study used an emotional Stroop task to investigate the existence of an attentional bias in math anxiety, the aim being to provide evidence for a possible mechanism by which math anxiety may originate, be maintained and/or become aggravated. In order to achieve this objective we designed an experiment that sought to overcome the methodological limitations that previous researchers had suggested that may have prevented them from observing the emotional Stroop effect in HMA individuals. The main methodological improvements were: (1) groups were formed according to extreme scores on math anxiety; (2) we used a computer-based task (like Hopko et al., 2002); (3) words were presented individually; (4) math-related words were carefully selected to be familiar for our sample; (5) several subject variables were controlled for; and (6) self-report measures were included in order to assess perceived anxiety toward each stimulus.

Our results showed that HMA individuals needed longer to report the ink color of math-related words as compared with neutral words, whereas no such difference emerged for their LMA counterparts. This difference shows that participants noticed the meaning of the irrelevant dimension of the task (i.e., stimulus content) and that this math-related content prolonged the time that HMA individuals needed to name the color in which the word was printed, as compared with a neutral one.

Previous research in other types of anxiety had already demonstrated the slow-down in the emotional Stroop task for those words related to the current concerns of the participant or patient. For example, this effect had been found for: physical threat words in panic disorder participants (Dresler et al., 2012), dentist-related words in high dental anxious subjects (Muris et al., 1995), social threat words for social phobics (Andersson et al., 2006), illness-related words in high health anxious individuals (Owens et al., 2004), physical threat words in somatoform patients (Lim and Kim, 2005), threat words (i.e., inept, ashamed) in people who stutter (Hennessey et al., 2014), cancer-related words in women with family histories of breast cancer (Erblich et al., 2003), etc. Our study extends these findings to the field of math anxiety.

However, what lies behind the delay in response times in the emotional Stroop task? Traditionally, the slowdown observed when comparing threatening vs. neutral information has been explained as an *attentional bias* toward threatening or emotional information (Williams et al., 1996). Nevertheless, the mechanisms underlying this attentional bias remain the subject of debate. In this respect, according to the *facilitated attention account*, emotional stimuli are noticed earlier than neutral stimuli (i.e., preferential engagement) and command attention at the expense of other stimuli or dimensions of the stimulus (i.e., ink color; Pratto and John, 1991; Williams et al., 1996). Consequently, the emotional Stroop effect is the product of the disproportionate amount of attention captured by emotional words, attention that would otherwise have been directed to performing the main task (i.e., naming the ink color). The *difficulty in disengagement account*, by contrast, argues that once attention is allocated toward a threat stimulus, it is held longer than in the case of neutral stimuli, thereby disrupting the processing of other stimulus properties and delaying the time needed to report the ink color (Fox et al., 2001).

Unfortunately, the emotional Stroop task does not allow us to distinguish which of these two components of attentional bias is responsible for the observed delay in response times. Thus, it could be the case that HMA individuals showed facilitated attention toward math-related content, such that the word “*fórmula”* (i.e., formula) captured more of their attention than did the word “*calzado”* (i.e., footwear), with the amount of attention that was drawn away from the main task causing the delay in response times. However, it is also possible that HMA individuals showed no preferential engagement but, rather, found it difficult to disengage their attention from math-related information, in which case the word “*fórmula”* would have held attentional resources for longer than did the word “*calzado”*, thereby explaining why they needed longer to respond to the former stimulus.

Further research is now needed to determine which of these two alternatives offers the best explanation for attentional bias in HMA individuals. A good option to this aim would be the *dot probe task* (see Rubinsten et al., 2015) in which two stimuli, one threat-related and the other one neutral are presented together in the same screen and their offset is followed by a small probe replacing one of the two stimuli, to which participants are instructed to respond. Trials can be congruent, if the dot replaces threat-related stimuli or incongruent, if the dot replaces a neutral one. One of the main advantages of this task is that, by including a control condition (i.e., two neutral stimuli presented together; Koster et al., 2004), researchers would be able to assess the different subcomponents of attentional bias.

Moreover, it is interesting to note that we found differences between math-anxious groups in a task requiring reporting the ink color of words, that is, a task involving no digits or numerical processing at all. This demonstrates that math anxiety can be raised by several types of stimuli, beyond numbers. In the same line, a previous study, using a novel priming task^3^, found that children with developmental dyscalculia (DD) responded faster to arithmetic equations that were presented after negative and math-related words, while the reverse pattern was shown by the control group (Rubinsten and Tannock, 2010). In other words, they found that simple arithmetic problem solving (i.e., addition, subtraction, multiplication and division) was modulated by math-related words (e.g., “quantity”) in the same way that in our study this type of words were related with slower response times, as compared with neutral ones, in a task requiring simply to report the ink color of words. In this line, while these two studies have used math-related words, it would be interesting to study math anxiety by means of other stimuli, such as pictures, which show the advantage of having more ecological validity, something that future studies should address.

To summarize, this study constitutes the first evidence showing an attentional bias toward math-related words in HMA individuals by means of an emotional Stroop task. Thus, it seems that Hopko et al. (2002) were right in their assumptions and that previous methodological limitations did prevent researchers from observing significant results in the past, reason why, after improving them, we finally were able to obtain significant differences between groups on attentional bias. Among these improvements, the fact of controlling participants’ level of trait anxiety was basic in order to rule out the possibility of general levels of trait anxiety explaining our results. In this respect, correlational analysis showed a very interesting result: while trait anxiety was related with slower response times both to math-related and neutral words, as well to with higher levels of self-reported anxiety toward both types of words, math anxiety showed a specific effect only for math-related words, being related with slower response times and with higher levels of self-reported anxiety toward them, but not relationship with neutral ones.

As commented earlier, this attentional bias toward math-related information may play a role in the origin, maintenance and/or aggravation of math anxiety. In this respect, it has been suggested that differences between low and high anxious individuals have to do with their responsiveness to minor threat cues that do not signal dangers requiring urgent action (Mathews and MacLeod, 2002). Thus, previous literature considers that there is a threat evaluation process in which a certain threshold must be exceeded in order to shift from a mode in which threat-related cues are ignored, to one in which they are attended. In this respect, it has been proposed that a lower threshold level (at which this shift takes place) may be associated with vulnerability to anxiety. Following this idea, it could be the case that children differ in this threshold determining if math-related information is ignored or attended. Thus, those children showing a tendency to easily exceed this threshold and attend to math-related information might be more vulnerable to develop math anxiety. Moreover, this favored attentional processing toward math-related stimuli would make HMA individuals overestimate the level of danger or the degree of threat in the environment (e.g., math class), leading to an increase in their level of math anxiety (i.e., heightened emotional reaction). This increase in their level of math anxiety would, in turn, contribute to a greater tendency to perceive math-related information as threatening, making them even more sensitive to their math concerns.

The fact of having found evidence for an attentional bias in math-anxious individuals can be very useful given that it constitutes the first step in order to set the path for the development of training programs aiming to correct it. For example, it has been shown that only one session of attention bias modification in subjects with social anxiety traits was sufficient to produce modifications in attention processing and vulnerability toward anxiety (Amir et al., 2009). Given the potential usefulness of investigating attentional bias in HMA individuals, future research deserves to be done in this line, in order to replicate the findings of this study by means of other experimental tasks, by further investigating which components of attentional bias might be mostly affected in HMA individuals and by trying to reveal the role of attentional control in this bias. Then, studies should be focused on proving the effectiveness of an attentional bias modification program in HMA individuals, both for avoiding the aggravation of math anxiety in those children who have started to show evidence of suffering from it, as well as for potentially reducing its negative impact on performance in those adults with a long history of math-anxiety.

## Conflict of Interest Statement

The authors declare that the research was conducted in the absence of any commercial or financial relationships that could be construed as a potential conflict of interest.

## Footnotes

1 In the dot probe task, neutral and threat-related stimuli are presented in the same screen and followed by a probe (a dot) to which the participant have to respond, and which appears either following the threat-related (congruent trials) or the neutral (incongruent trials) stimulus. Thus, while in the classical version of this task the dot appears with the same frequency in each location (i.e., same number of congruent and incongruent trials), in the dot probe training procedure this contingences between stimulus and probe positions are arranged in order to induce a temporary attentional bias either toward or away from threat-related information.

2 The Spanish lexical database defined word frequency as the number of times the word appears in the EsPal corpus divided by the total count of the EsPal corpus words multiplied by one million. Familiarity, imageability and concreteness were assessed by means of the questions: ‘*How familiar are you with this word on a scale of 1 to 7, with 7 being most familiar?’, ‘How imageable is this word on a scale of 1 to 7, with 7 being the most imageable?’, and ‘How concrete is this word on a scale of 1 to 7, with 7 being most concrete?’, respectively..*

3 In this task, people typically respond to target stimuli more quickly after presentation of an affectively related prime than after one that is unrelated affectively.
