# Supplementary material for: Attentional bias in high math-anxious individuals: evidence from an emotional Stroop task
Source: Front Psychol. 2015 Oct 19;6:1577. doi: 10.3389/fpsyg.2015.01577 (PMC4609828; doi:10.3389/fpsyg.2015.01577)
Supplement: Supplementary file 1 [file Data_Sheet_1.DOCX]

**Appendix**

| Spanish Word | English translation | Frequency | Number of phonemes | Familiarity | Imageability | Concreteness |
| --- | --- | --- | --- | --- | --- | --- |
| Neutral words presented in the training session | | | | | | |
| Jungla | Jungle | 3.57 | 6 | 5.35 | 6.22 | 4.96 |
| Jugador | Player | 97.00 | 7 | 5.95 | 5.97 | 4.76 |
| Máquina | Machine | 37.93 | 6 | 5.98 | 5.56 | 4.24 |
| Camino | Path | 209.90 | 6 | 5.97 | 6.10 | 4.75 |
| Empresa | Company | 164.22 | 7 | 5.84 | 5.07 | 5.17 |
| Math-related words presented in the experimental session | | | | | | |
| Álgebra | Algebra | 4.41 | 7 | - | - | - |
| Cálculo | Calculus | 23.95 | 7 | 5.01 | 3.34 | 4.04 |
| Ecuación | Equation | 13.17 | 8 | 5.08 | 5.61 | 4.85 |
| Matemáticas | Mathematics | 20.92 | 11 | 5.62 | 4.75 | 5.21 |
| Estadística | Statistics | 18.06 | 11 | 5.71 | 3.3 | 4.36 |
| Fórmula | Formula | 42.21 | 7 | 4.97 | 4.64 | 4.25 |
| Geometría | Geometry | 10.90 | 9 | 3.89 | 3.30 | 4.50 |
| Logaritmo | Logarithm | 0.65 | 9 | - | - | - |
| Multiplicación | Multiplication | 5.60 | 14 | - | - | - |
| Número | Number | 341.66 | 6 | 6.50 | 6.48 | 5.04 |
| Resta | Subtraction | 6.10 | 5 | 5.98 | 3.92 | 4.94 |
| Suma | Addition | 53.12 | 4 | 5.91 | 4.7 | 4.84 |
| División | Division | 123.19 | 8 | 5.40 | 5.34 | 4.16 |
| Calculadora | Mathematical calculator | 1.75 | 11 | - | - | - |
| Neutral words presented in the experimental session | | | | | | |
| Silueta | Silhouette | 4.48 | 7 | 4.09 | 5.88 | 4.92 |
| Respaldo | Back | 24.21 | 8 | 5.39 | 4.66 | 5.09 |
| Inscripción | Registration | 13.42 | 11 | 5.72 | 4.95 | 4.89 |
| Empresario | Entrepreneur | 21.27 | 10 | 5.41 | 5.70 | 5.19 |
| Funcionario | Civil servant | 19.14 | 11 | 5.43 | 4.80 | 4.57 |
| Patria | Homeland | 42.05 | 6 | 4.49 | 3.95 | 4.98 |
| Trayecto | Journey | 10.91 | 8 | 5.22 | 4.48 | 4.68 |
| Recibidor | Reception | 0.71 | 9 | 5.17 | 5.05 | 4.46 |
| Sugerencia | Suggestion | 5.80 | 11 | 4.24 | 4.42 | 4.56 |
| Población | Population | 324.75 | 9 | 5.77 | 4.91 | 4.41 |
| Calzado | Footwear | 6.13 | 7 | 5.99 | 5.77 | 5.03 |
| Ruta | Route | 54.20 | 4 | 5.82 | 5.43 | 5.0 |
| Reforma | Refurbishment | 123.05 | 7 | 6.10 | 4.75 | 4.21 |
| Simulacro | Simulation | 1.55 | 9 | 4.25 | 4.55 | 4.56 |
